# Effects of titanium mesh implant on dosimetry during Gamma Knife radiosurgery

**DOI:** 10.1120/jacmp.v13i5.3833

**Published:** 2012-09-06

**Authors:** Joseph T. Rakowski, Karen Chin, Sandeep Mittal

**Affiliations:** ^1^ Departments of Radiation Oncology Karmanos Cancer Institute Wayne State University Detroit Michigan USA; ^2^ Departments of Neurosurgery Karmanos Cancer Institute Wayne State University Detroit Michigan USA

**Keywords:** stereotactic radiosurgery, titanium cranioplasty, radiosurgery dosimetry, GAFCHROMIC film

## Abstract

Calvarial reconstruction following resection of tumors involving the skull is often followed by stereotactic radiosurgery. Prior studies have addressed the effects of various cranioplasty materials on dose distributions in linac‐based radiosurgery. We aim to determine the effects of titanium mesh implants on Gamma Knife dose. Radiation backscatter and transmission were measured for eight types of titanium mesh using film, ion chamber, and Theratron Co‐60 teletherapy device. A single mesh was selected for Gamma Knife irradiation using a ${\text{CaSO}}_{4}$ skull filled with ballistics gel. Dose profiles for reconstructed and intact skulls were compared with the planning system prediction at 2.5 and 5.5 cm depth. Titanium contact backscatter and transmission dose perturbations ranged from ‐18% to 23%. Radiation dose measured at 1.5 cm below the calvarial implant increased by 0.5% to 3.3% relative to bone. Measured Gamma Knife dose profile diameters agreed with expected profiles. Maximum dose within the intact phantom was 3% less than planned due to skull attenuation. Maximum dose within the reconstructed phantom was between the intact phantom and planned doses. Titanium mesh implants and hydroxyapatite cranioplasty result in minimal alteration (<3%) in the delivered Gamma Knife dose.

PACS number: 87.00

## I. INTRODUCTION

Reconstruction of the calvarium with thin titanium mesh implants (≤ 0.7 mm thickness) after neurosurgical intervention can be followed by stereotactic radiosurgery (SRS). A popular radiosurgery system is the Gamma Knife (GK), using 201 focused Co‐60 sources. The sources are arranged in five concentric rings with the axes of all source beamlets focused to a single point. The Co‐60 energy of 1.25 MeV is lower than that of linear accelerator (linac)‐based systems, thereby creating differences in amount and types of interactions in metallic cranial implants or devices relative to linac energies. A few publications have addressed the effects of thin titanium mesh implants on dose distributions in linac‐based radiosurgery.^1^, ^2^ Several other investigators have studied perturbation of Co‐60 and linac‐based radiotherapy dose from thicker metallic materials such as aneurysm clips and coils,^3^, ^5^ as well as head and neck reconstruction devices.^6^, ^14^ However, there are at present no studies evaluating the effects of cranial metallic implants on dose distributions in GK SRS.

## II. MATERIALS AND METHODS

Transmission and backscatter radiation properties of several titanium meshes, hydroxyapatite bone cement, ${\text{CaSO}}_{4}$ phantom skull material, and a sample of human skull were evaluated using a Theratron Co‐60 teletherapy unit (Best Theratronics, Ottawa, ON, Canada), ballistics gel, solid water, GAFCHROMIC film (International Specialty Products EBT),^(15)^ and ion chamber. A head phantom was constructed to test the effects of the various cranioplasty matierials on the GK dose distribution. The phantom comprised a simulated skull made of plaster $\left({\text{CaSO}}_{4}\right)$, tissue equivalent ballistics gel to simulate soft tissues, and a simulated skin layer made of a 3 mm layer of Aquaplast (Qfix, Avondale, PA). GK dose distributions were measured using GAFCHROMIC film (International Specialty Products MD‐55^(16)^). All films were scanned on an Epson model 10000 XL flatbed transmission scanner and analyzed with Radiological Imaging Technology Incorporated (Radiological Imaging Technology, Colorado Springs, CO) film analysis software.

**Figure 1:**
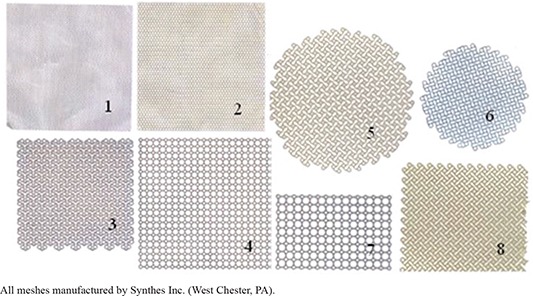
The eight different meshes evaluated in this study. Dimensions: 1. 10×10 cm t: 0.14 mm; 2. 10×10 cm t: 0.25 mm; 3. 9.2×8.9 cm t: 0.64 mm; 4. 10×10 cm t: 0.51 mm; 5. d: 10.1 cm t: 0.6 mm; 6: d: 7.35 cm t: 0.4 mm; 7. 5.3×8 cm t: 0.7 mm; 8. 7.3×15 cm t: 0.7 mm.

Eight different titanium neurosurgical meshes (Synthes Inc., West Chester, PA), distinguishable by thickness and hole pattern (Fig. 1), were tested. The transmission and backscatter properties of the eight meshes and 0.5 cm thick hydroxyapatite bone cement were measured in the Co‐60 teletherapy unit using a single Co‐60 beam collimated to a $3\times 3{\;\text{cm}}^{2}$ field using a cerrobend block. Contamination of the beam by leakage and scattered photons was eliminated by surrounding the test cell with lead bricks. A dose of 110 cGy was delivered in a single fraction. Beam profiles were measured in tissue equivalent ballistics gel with EBT film in contact with the top and bottom surfaces of the meshes and a 0.5 cm layer of ballistics gel on the top surface.

Ion chamber measurements of transmission through the metallic implants, ${\text{CaSO}}_{4}$ skull phantom material, and resected skull sample were done in solid water at a depth of 1.5 cm beneath the titanium implant, ${\text{CaSO}}_{4}$, and bone. Mesh #1 was selected for GK irradiation because it provided the greatest transmission, as demonstrated in Table 1.

**Table 1:** Ion chamber transmission measurement ratios.

*Mesh*	$Alloplast/CasO_{4}$	*Alloplast/Bone*
1	1.033	1.033
2	1.025	1.025
3	1.026	1.026
4	1.020	1.019
5	1.027	1.027
6	1.032	1.032
7	1.026	1.026
8	1.026	1.026
0.5 cm hydroxyapatite	1.005	1.005

The head phantom was fixed in the Leksell stereotactic frame and imaged with a volumetric CT scan in preparation for treatment planning. Two plans were created in the Leksell

GammaPlan 8.3 software (Elekta AB, Stockholm, Sweden). The first plan consisted of a single 18 mm diameter shot with the isocenter at 2.5 cm depth beneath the skin surface treated at 9 Gy to the 50% isodose line. The second plan had the same parameter except for a deeper isocenter, 5.5 cm instead of 2.5 cm.

**Figure 2:**
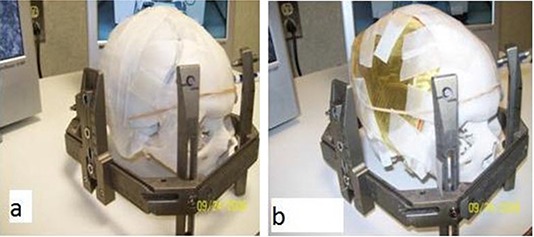
Intact skull phantom in Leksell frame (a) and resected with mesh and gel (b).

Each plan was evaluated with two different experimental setups: with an intact skull, and a resected skull with the resected section replaced with the mesh covered with a 5 mm thick layer of gel (Fig. 2). For radiation transmission purposes, the Leksell GK planning software assumes the head is equivalent to water and does not correct for bone or air heterogeneities. The MD‐55 film was positioned in the coronal plane of the phantom. Superior–inferior and right–left orthogonal profiles were analyzed.

## III. RESULTS

**Figure 3:**
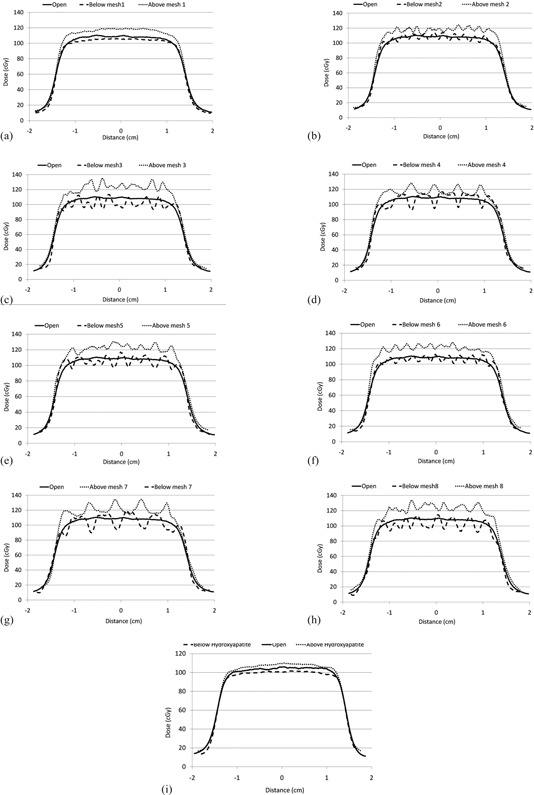
Mesh profiles ((a)‐(i)) immediately above and below the mesh compared to the open field for eight different titanium meshes and 0.5 cm hydroxyapatite.

The beam profiles for the eight meshes and hydroxyapatite bone cement measured in the Co‐60 Theratron unit, with film in contact with the cranioplasty implant, are presented in (Figs. 3a)‐(i). Profiles of the open field are compared to the implant profiles. Backscattered radiation dose perturbation is demonstrated in the profiles above the mesh. The amount and pattern of backscatter varied among the different types of meshes. The patterns blend into a uniform distribution within 5 mm of the mesh surface.

Dose above the cranial implant varied in the valleys and peaks from 120 to 135 cGy, which corresponds to a 9% to 23% increase in dose relative to an open field. Beneath the implants, the dose varied across the profile from a minimum of 90 cGy to a maximum of 115 cGy, approximately an 18% decrease and a 4.5% increase in dose under the mesh relative to an open field, respectively. Hydroxyapatite backscatter and transmitted dose perturbation were approximately +5% and ‐5%, respectively. In comparison, the Co‐60 backscatter dose perturbation factor at the surface of bone is 8.5%.^(17)^

Profile patterns vary depending on the mesh hole patterns, diameters, and material thickness. Some profiles are smoother such as profiles from mesh #1, #2, and #6, which are thinner with denser mesh patterns and smaller holes. The thicker meshes with larger holes demonstrated greater amounts of dose perturbation. The patterns also can vary with position of the measurement line across the film image.

Table 1 summarizes the ion chamber results for the eight meshes and hydroxyapatite, relative to the ${\text{CaSO}}_{4}$ section and bone. The greatest transmission through the implant occurs for mesh #1, with a ratio of 1.033. The least transmission occurs for hydroxyapatite with a ratio of 1.005.

**Figure 4:**
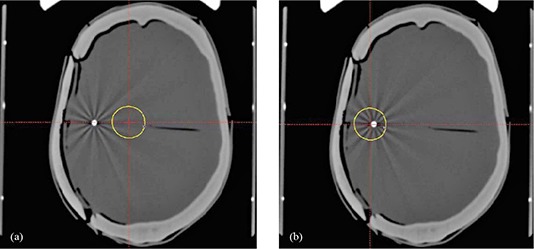
Treatment planning axial CT slices through intact skull for 5.5 cm depth (a) and 2.5 cm depth (b).

**Figure 5:**
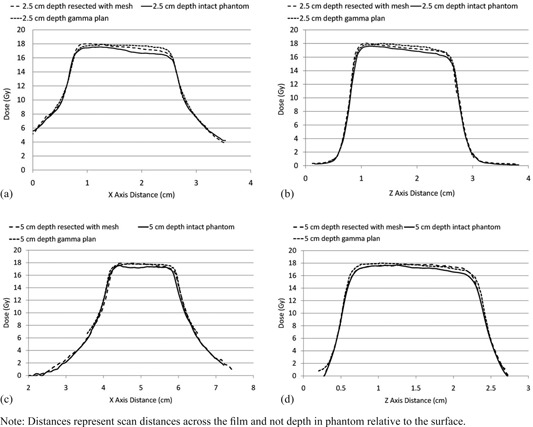
2.5 and 5 cm depths x‐ and z‐axis coronal plane dose profiles ((a)‐(d)) from Leksell Gamma Knife treatment plan, measured dose profile for unresected phantom, and measured dose profile for resected with mesh.

Mesh #1 was selected for subsequent GK delivery as it provided the greatest transmission relative to bone (Table 1). Treatment planning axial CT images of the intact skull with the 50% isodose line are shown in (Figs. 4a) and (b) for the 5.5 cm depth and 2.5 cm depth, respectively. The resulting GK dose profiles in the coronal plane for the 2.5 and 5.5 cm deep isocenters are shown in (Figs. 5a)–(d). Each plot shows the dose profiles with the intact CaSO4 phantom skull, the resected phantom with implanted titanium mesh, and the line dose profiles from the Leksell GammaPlan treatment planning software. The measured versus planned profile shapes agree along the x‐ (right–left) and z‐ (inferior–superior) axes. The maximum dose in the intact phantom was reduced approximately 3%. This agrees with Gamma Knife Monte Carlo calculations using a water and bone phantom model.^(18)^ The Leksell GammaPlan does not correct for skull heterogeneities, but assumes water throughout. The maximum dose in the resected phantom lies between the intact phantom maximum dose and planned dose. This is due to increased transmission through the titanium mesh relative to ${\text{CaSO}}_{4}$. Agreement between the measured and planned 50% isodose diameters along the x‐axis were within 1 mm for the 2.5 cm depth and within 0.3 mm for the 5 cm depth. Along the z‐axis, the 50% isodose diameters agreement was within 0.3 mm for both depths.

## IV. DISCUSSION

Backscattered and transmitted dose perturbations are pronounced at the surface of the titanium mesh, and to a lesser degree with the hydroxyapatite cranioplasty. Backscatter dose perturbation ranged from 9% to 23% of the single beam open field dose. Transmitted dose perturbation ranged from ‐18% to 4.5% of the single beam open field dose. The dose perturbations are generated through Compton interactions of the Co‐60 primary photons in the mesh producing secondary electrons, and interactions in the mesh of the secondary electrons generated upstream in tissue by the primary photons. The profiles are influenced by the hole diameters and patterns of the titanium meshes. The amount of interaction and resulting perturbation increases with mesh thickness, as demonstrated by the profiles in Fig. 3. The dose pattern diminishes to a smooth profile within 5 mm distance from the mesh. Hydroxyapatite backscatter and transmission properties were similar to bone.

As outlined in Table 1, equality of the transmission measurement ratios of implant/${\text{CaSO}}_{4}$ and implant/bone suggest that the radiologic properties of the ${\text{CaSO}}_{4}$ skull phantom are very similar to those of true skull at Co‐60 energies. The titanium mesh is less attenuating than true bone by no more than 3.3%. This did not have a significant impact on GK treatment delivery since the GK planning system does not account for the approximate 3% attenuation in the skull, and the resected area intersected approximately 50 of the 201 beams in the Leksell GK model 4C. Thus replacing skull with a cranial implant raises the delivered dose closer to the planned dose.

Gamma Knife x‐ and z‐axis dose profiles measured in the skull phantom agree with the planning system profiles, with slight deviations caused by the radiation attenuation properties of the ${\text{CaSO}}_{4}$ skull and mesh. The approximate 3% reduction in maximum dose with the intact skull agrees with Monte Carlo predictions.^(18)^ Replacement of the resected section of skull with the titanium mesh allowed approximately 3.3% additional radiation transmission through that section which raised the measured dose profiles closer to the GammaPlan generated profiles.

The GAFCHROMIC film manufacturer specifies the MD‐55 single sheet sensitometric response uniformity 95% confidence as <8%, and sheet‐to‐sheet sensitometric response uniformity 95% confidence as <5%, for a quadrature sum of < 9.4%.^(16)^ The dose uncertainties corresponding to ±9.4% sensitometric response for this particular film calibration are approximately ±6.5% in the 35 Gy to 40 Gy range, and ±18% in the 8 Gy to 12 Gy range. Uncertainty variation with dose is due to the dose dependence of the calibration curve slope. This uncertainty is greater than the differences in maximum dose between the measured and planned profiles. Consistency in the profiles and agreement with Monte Carlo skull attenuation studies suggest the film performed well within these specifications at doses around 18 Gy. Differences between measured and planned 50% isodose diameters can be explained by a ±5% dose uncertainty around 9 Gy.

Overall, the results suggest that titanium mesh implants and hydroxyapatite cranioplasty result in minimal alteration (<3%) in the delivered GK dose. This is the first report to address the effects of titanium and nonmetallic implants on dose distributions in Gamma Knife radiosurgery.

## V. CONCLUSION

Gamma Knife cranial SRS performed on patients with titanium mesh implants or hydroxyapatite cranioplasty does not compromise successful delivery of the prescribed isodose. Replacement of resected calvarium with titanium mesh serves to raise the delivered maximum dose closer to the planned maximum dose, due to the absence of heterogeneity corrections in the Gamma Knife dose calculation algorithm. The radiation attenuation properties of hydroxyapatite at Co‐60 energies are very similar to cranial bone, thereby leaving the delivered isodose volume unaltered from that attained with an intact calvarium. Backscattered and transmitted dose perturbations are pronounced at the surface of the titanium mesh and, to a lesser degree, with the hydroxyapatite cranioplasty. Titanium backscatter dose perturbation ranged from 9% to 23% of the single beam open field dose. Titanium transmitted dose perturbation ranged from ‐18% to 4.5% of the single beam open field dose. Hydroxyapatite backscatter and transmitted dose perturbation were approximately +5% and ‐5%, respectively. In comparison, the Co‐60 backscatter dose perturbation factor at the surface of bone is 8.5%.
